# Genome sequence of Shiga toxin-producing *Escherichia coli* jumbo bacteriophage vB_EcoM_JNE01

**DOI:** 10.1128/mra.01145-23

**Published:** 2024-01-24

**Authors:** Xiaoqian Wang, Xiaotian Wei, Qing Zhang, Lulu Li, Zhengjie Liu, Yibao Chen, Yuqing Liu, Yumei Cai

**Affiliations:** 1 College of Veterinary Medicine, Shandong Agricultural University, Taian, China; 2 Shandong Key Laboratory of Animal Disease Control and Breeding, Institute of Animal Science and Veterinary Medicine, Shandong Academy of Agricultural Sciences, Jinan, China; 3 Key Laboratory of Livestock and Poultry Multi-omics of MARA, Jinan, China; Queens College Department of Biology, USA

**Keywords:** bacteriophages, genomes

## Abstract

Bacteriophage vB_EcoM_JNE01 was isolated from chicken farm sewage using *Escherichia coli* O157:H7 as the host bacteria. The total length of the vB_EcoM_JNE01 genome is 355,583 bp, with 584 open reading frames and 36% G+C content. It shares an 80% nucleotide identity with 59% query coverage with the bacteriophage PBECO4 (NC_027364).

## ANNOUNCEMENT

Shiga toxin-producing *Escherichia coli* (STEC) O157:H7 is a zoonotic pathogen of wide-host animals that can be transmitted through a variety of media, including food and water, and poses a significant threat to human health and food safety (1).

In June 2023, we isolated the jumbo phage vB_EcoM_JNE01 from chicken farm sewage using STEC O157:H7 as the host bacteria. Briefly, 50 mL of wastewater was collected from a farm in Jinan, China (36°40′N, 117°00′S) and then sterilized by filtration through a 0.22-µm membrane. The conventional double-layer agar method was used to examine phage activity as previously described (2–4). One of the single plaques was picked aseptically and re-suspended in 5 mL SM buffer (100 mmol/L NaCl, 8 mmol/L MgSO_4_, 0.05 mol/L Tris-HCl, and 0.01% gelatin; pH 7.4–7.6). Phage particles, vB_EcoM_JNE01, were purified by CsCl gradient ultracentrifugation and observed with a transmission electron microscope (JEM-1400Flash, Japan). Under electron microscope observation, vB_EcoM_JNE01 has an isometric head (122 ± 3 nm in diameter) and a contractile tail (132 ± 4 nm long and 20 ± 1 nm wide) (data not shown).

Phage vB_EcoM_JNE01 DNA was extracted using the λ phage genome rapid extraction kit (ZOMANBIO). Following the use of 100 ng of DNA in the NEBNext Ultra II FS DNA Library Prep Kit, DNA was quantified using a QubitTM fluorometer (NEB). According to the Annoroad Universal DNA Fragmentase Kit V2.0 protocol, DNA samples were combined with 10× FD, fragmentase, and other reagents for DNA fragmentation, resulting in ~300-bp fragments. The fragment ends were subsequently repaired, and TruSeq adapters were added using the Annoroad Universal DNA Library Prep Kit v2.0. The Agilent 2100 Bioanalyzer system was utilized to detect the scattering of library fragments along with KAPA Library Quantification Kits. Complete genome sequencing of phage vB_EcoM_JNE01 was performed using the NovaSeq 6000 system. Sequencing generated 7,334,589 single-end total reads with 2 × 150 bp read length, and the average sequencing depth was 46.5×. Adapter sections and low-quality reads were removed by means of Trimmomatic 0.36 at default parameters. The raw reads were assembled using SPAdes v.3.13.0. Phage vB_EcoM_JNE01 genome was annotated using RAST (http://rast.nmpdr.org/). Protein BLAST (https://www.ncbi.nlm.nih.gov/BLAST/) was used to identify putative homology and functions of predicted phage proteins. VirulenceFinder—VFDB (https://cge.cbs.dtu.dk/services/VirulenceFinder/, accessed on 29 June 2021) and ResFinder 4.1 (https://cge.cbs.dtu.dk/services/ResFinder/) determined the virulence and antibiotic resistance genes. Phylogenetic analysis of phage large terminase subunit sequences, which was downloaded from the NCBI database, was performed using the ClustalW program in MEGA 11 (5), and phylogenetic tree was generated using the neighbor-joining method and 1,000 bootstrap replicates.

**Fig 1:**
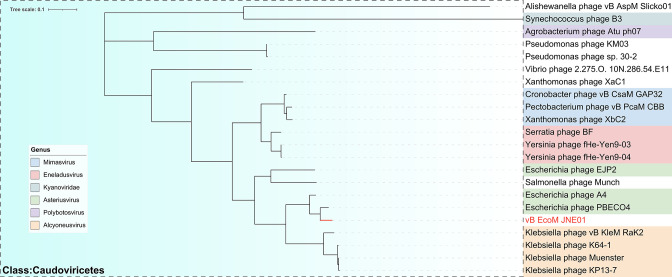
Phylogenetic relationships between the terminase large subunit (ORF548) of phage vB_EcoM_JNE01 and other phages belonging to the order Caudovirales. The phylogenetic tree was generated using the neighbor-joining method and 1,000 bootstrap replicates.

Phage vB_EcoM_JNE01 possesses a double-stranded genome of 355,583 bp along with 36% of GC content. A total of 584 open reading frames (ORFs) were found in the genome of phage vB_EcoM_JNE01, of which 138 (23%) had predicted functions. Phage vB_EcoM_JNE01 genome did not contain any virulence or antibiotic resistance genes. It shares 80% nucleotide identity with 59% query coverage with the phage PBECO4 (GenBank no. NC_027364). Phylogenetic tree analysis of phage-encoded terminase large subunit (ORF548) revealed a close phylogenetic relationship between phage vB_EcoM_JNE01 and PBECO4 (Fig. 1), suggesting that they all belong to Asteriusvirus genus.

## Data Availability

The complete genome sequence of phage vB_EcoM_JNE01 accession no. OR670491, SRA accession no. SRR26539804, and BioSample accession no. SAMN37954867.
